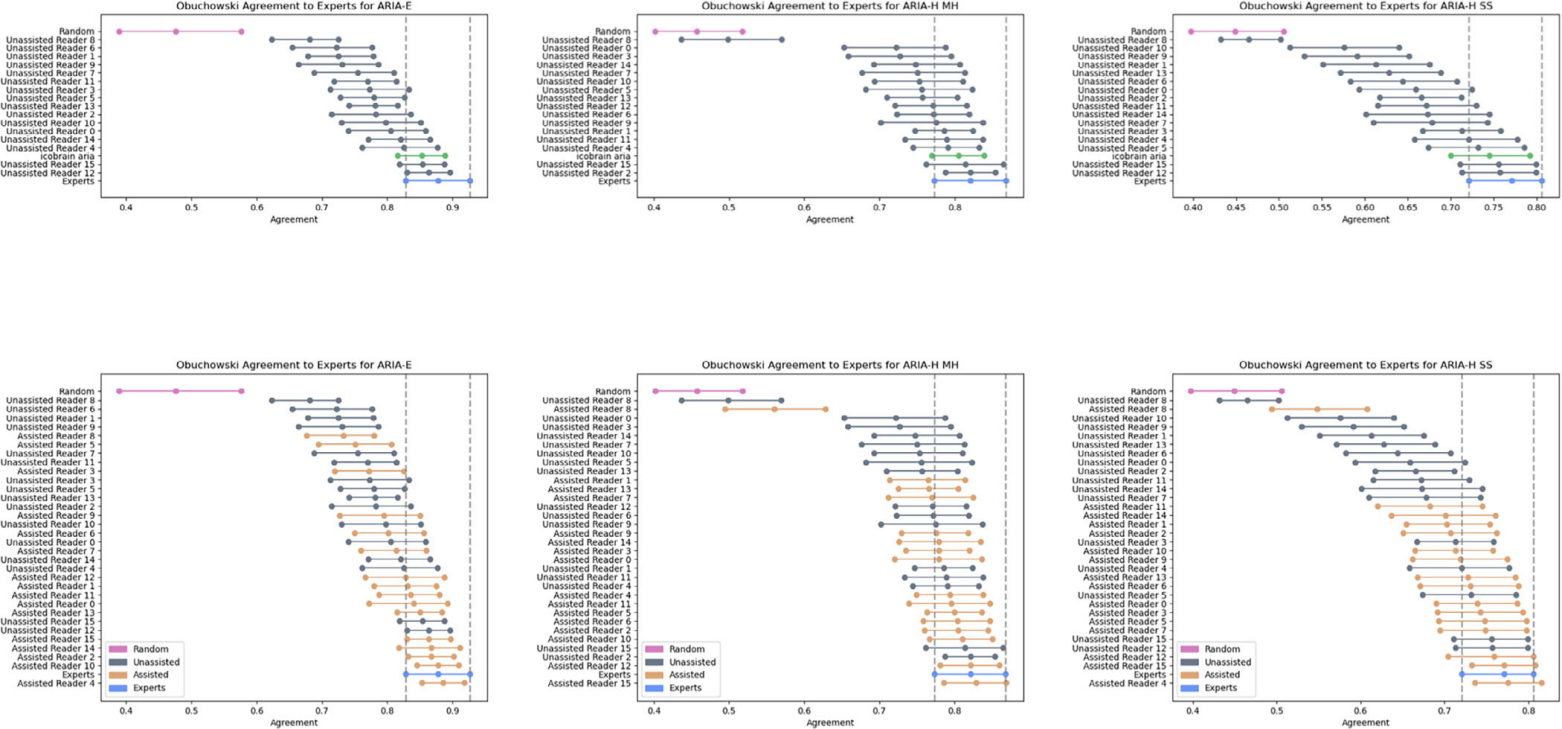# Assessing the impact of automated assistive automated detection and severity grading software on ARIA reading

**DOI:** 10.1002/alz70856_100401

**Published:** 2025-12-25

**Authors:** Arno Liseune, Ricardo Magalhães, Simon Van Eyndhoven, Thanh Van Phan, Arne Brys, Rafay A Khan, Wim Van Hecke, Annemie Ribbens, Diana M. Sima, Dirk Smeets

**Affiliations:** ^1^ Icometrix, Leuven, Belgium

## Abstract

**Background:**

Amyloid beta‐directed monoclonal antibody therapies may cause ARIA‐E (edema) and ARIA‐H (microhemorrhage (MH) and superficial siderosis (SS)). Studies have shown that clinicians find it challenging to identify or evaluate ARIA. As treatment decisions are informed by ARIA presence and severity, assistive automated detection and severity grading software may significantly improve radiological reading.

**Method:**

ICO**brain aria** is the first FDA‐cleared CADe/CADx software for automated detection and severity grading of ARIA‐E and ARIA‐H. Sixteen non‐ARIA‐expert radiologists evaluated ARIA in 199 cases of Alzheimer's disease patients treated with aducanumab. They read the cases twice, i.e., assisted and unassisted by ico**brain aria**. Ground truth ARIA severity was established by three expert neuroradiologists for ARIA‐E, ARIA‐H MH and ARIA‐H SS and was compared between the standalone software and experts, and between (un)assisted radiologists and experts using the Obuchowski agreement metric.

**Result:**

The ARIA severity grading suggested automatically by the software in the sample of 199 cases was interchangeable with experts, meaning that its agreement to experts was at the same level as inter‐expert agreement. Indeed, the Obuchowski agreement metric between the 3 experts was 0.878 [CI: 0.828, 0.927] for ARIA‐E, 0.821 [CI: 0.773, 0.868] for ARIA‐H MH and 0.771 [CI: 0.721, 0.806] for ARIA‐H SS, while ico**brain aria**'s was 0.853 [CI: 0.816, 0.889] for ARIA‐E, 0.805 [CI: 0.769, 0.839] for ARIA‐H MH and 0.745 [0.7, 0.792] for ARIA‐H SS. See Figure 1 top.

The interchangeability of non‐expert radiologists with experts increased significantly (Wilcoxon test *p*‐value<0.05) from 0.78 ± 0.05, 0.75 ± 0.07, 0.66 ± 0.07 for the unassisted readers to 0.83 ± 0.05, 0.78 ± 0.06, 0.72 ± 0.05 for the assisted readers for ARIA‐E, ARIA‐H MH and ARIA‐H SS, respectively (Figure 1 bottom).

**Conclusion:**

The standalone software ico**brain aria** grades ARIA severity closer to experts than unassisted radiologists do, but when using the software as an assistive tool, radiologists grade ARIA severity more interchangeably with experts.